# Induced pluripotent stem cell generation from a man carrying a complex chromosomal rearrangement as a genetic model for infertility studies

**DOI:** 10.1038/srep39760

**Published:** 2017-01-03

**Authors:** Aurélie Mouka, Vincent Izard, Gérard Tachdjian, Sophie Brisset, Frank Yates, Anne Mayeur, Loïc Drévillon, Rafika Jarray, Philippe Leboulch, Leila Maouche-Chrétien, Lucie Tosca

**Affiliations:** 1 AP-HP, Service d’Histologie, Embryologie et Cytogénétique, Hôpitaux Universitaires Paris-Sud, Hôpital Antoine Béclère, 92140, Clamart, France; 2 Université Paris-Sud, 94276 Le Kremlin-Bicêtre cedex, France; 3 AP-HP, Service de Gynécologie-Obstétrique et Médecine de la Reproduction, Hôpitaux Universitaires Paris-Sud, Hôpital Antoine Béclère, 92140, Clamart, France; 4 Sup’Biotech Villejuif 94800, Commissariat à l’Energie Atomique et aux Énergies Alternatives, Institute of Emerging Diseases and Innovative Therapies (iMETI), SEPIA, 92265 Fontenay-aux-Roses, France; 5 Commissariat à l’Energie Atomique et aux Énergies Alternatives, Institute of Emerging Diseases and Innovative Therapies (iMETI), 92265 Fontenay-aux-Roses; UMR-E 007, Université Paris-Saclay, 91400 Orsay; INSERM, 75013 Paris, France

## Abstract

Despite progress in human reproductive biology, the cause of male infertility often remains unknown, due to the lack of appropriate and convenient *in vitro* models of meiosis. Induced pluripotent stem cells (iPSCs) derived from the cells of infertile patients could provide a gold standard model for generating primordial germ cells and studying their development and the process of spermatogenesis. We report the characterization of a complex chromosomal rearrangement (CCR) in an azoospermic patient, and the successful generation of specific-iPSCs from PBMC-derived erythroblasts. The CCR was characterized by karyotype, fluorescence *in situ* hybridization and oligonucleotide-based array-comparative genomic hybridization. The CCR included five breakpoints and was caused by the inverted insertion of a chromosome 12 segment into the short arm of one chromosome 7 and a pericentric inversion of the structurally rearranged chromosome 12. Gene mapping of the breakpoints led to the identification of a candidate gene, *SYCP3*. Erythroblasts from the patient were reprogrammed with Sendai virus vectors to generate iPSCs. We assessed iPSC pluripotency by RT-PCR, immunofluorescence staining and teratoma induction. The generation of specific-iPSCs from patients with a CCR provides a valuable *in vitro* genetic model for studying the mechanisms by which chromosomal abnormalities alter meiosis and germ cell development.

Infertility is a complex disorder affecting 10 to 15% of couples of reproductive age worldwide^1^,^2^. Male infertility is the cause in about half the affected couples, and in 20% of cases, abnormalities are found only in the man. Most commonly, male infertility is associated with a defect of sperm parameters in terms of quantity and mobility (oligoasthenozoospermia). At worst, there are no spermatozoa in semen, a condition known as azoospermia. Azoospermia may be obstructive due to anatomical problems, infectious disease or vasectomy, or non-obstructive due to genetic defects, hormonal imbalance and environmental factors. However, a large proportion of affected men have idiopathic infertility, reflecting our poor understanding of the basic mechanisms regulating spermatogenesis.

Spermatogenesis is a complex process by which haploid gametes are formed through a specific type of cell division called meiosis. Meiotic abnormalities can occur at any stage of maturation and frequently contribute to reproductive failure. About 10% of infertile men display a major impairment of sperm production^3^.

Chromosomal abnormalities, including aneuploidies, deletions, microdeletions of the AZF region of the Y chromosome, duplications, small supernumerary marker chromosomes, translocations, insertions, and inversions, may be the underlying cause of the failure to complete meiosis^4^,^5^,^6^,^7^,^8^,^9^,^10^,^11^,^12^,^13^,^14^,^15^,^16^. Such chromosomal aberrations have been shown to affect meiotic synapsis and chromosome pairing^17^, resulting in both infertility and spontaneous abortions. However, despite the great insight into the genetics of infertility provided by conventional and molecular cytogenetic tools, our knowledge of the genetic causes of male infertility remains limited.

Various models have been developed to explain the relationship between chromosome mismatching during meiosis and spermatogenesis alterations^18^. A correlation between poor chromosome pairing or a complete absence of pairing and gametogenesis failure has been demonstrated in the *Drosophila* model^18^. Furthermore, changes in the expression of essential genes may account for spermatogenesis defects. Studies of male reproductive function in humans are hindered by the lack of human testicular specimens, which are not easy to obtain. Targeted mutagenesis in mice is the strategy most widely used for the modeling of meiosis abnormalities and for analyses of spermatogenesis failure and investigations of the causes of male infertility^19^. Studies of mouse models have led to the identification of 388 genes involved in spermatogenesis, but their relevance to human infertility remains to be demonstrated^20^.

Advances in cytogenetics and molecular biology have led to the identification of candidate genes in humans on the basis of the phenotype observed in knockout mouse models^20^. However, despite substantial efforts, the etiology and genetic causes of spermatogenetic failure remain unexplained in a large proportion of infertile men.

Techniques for reprogramming adult cells to readopt a pluripotent state have paved the way for a new era of disease modeling^21^,^22^,^23^,^24^,^25^,^26^. Indeed, the induced pluripotent stem cell (iPSC) technology pioneered by Shinya Yamanaka in 2006 has potential for unlimited expansion and can generate the cells of all three germ layers^27^. This is an essential property for research on diverse diseases and adjustments to treatment, opening up many new treatment possibilities^28^,^29^. Patient-specific iPSCs, with the same genetic background as the donor cells, provide unprecedented human models for studying genetic disorders^30^. Induced pluripotent stem cells have recently been generated from the fibroblasts of azoospermic men carrying deletions in the AZF region^31^ and from patients with Klinefelter syndrome (47, XXY)^32^,^33^.

We describe here the case of an azoospermic patient carrying a *de novo* complex chromosomal rearrangement (CCR). This patient was found to have a two-step CCR with five breakpoints due to the inverted insertion of a chromosome 12 segment into the short arm of one chromosome 7 and a pericentric inversion of the structurally rearranged chromosome 12. We identified *SYCP3* (synaptonemal complex protein 3) as a potential candidate gene responsible for his infertility. Through non-invasive primary cell collection and the use of non-integrative Sendai viruses expressing the human *OCT4, SOX2, KLF4* and *C-MYC* genes, we generated patient-specific iPSC lines to model this CCR for future studies. The pluripotency of the iPSCs was confirmed by assessing the expression of pluripotency markers and teratoma formation after injection of the cells into immunodeficient mice. The iPSCs derived in this study constitute the first genetic model for *in vitro* studies of the mechanisms by which chromosomal aberrations affect spermatogenesis in humans ever described.

## Results

### Conventional and molecular characterization of the CCR

**Figure 1:**
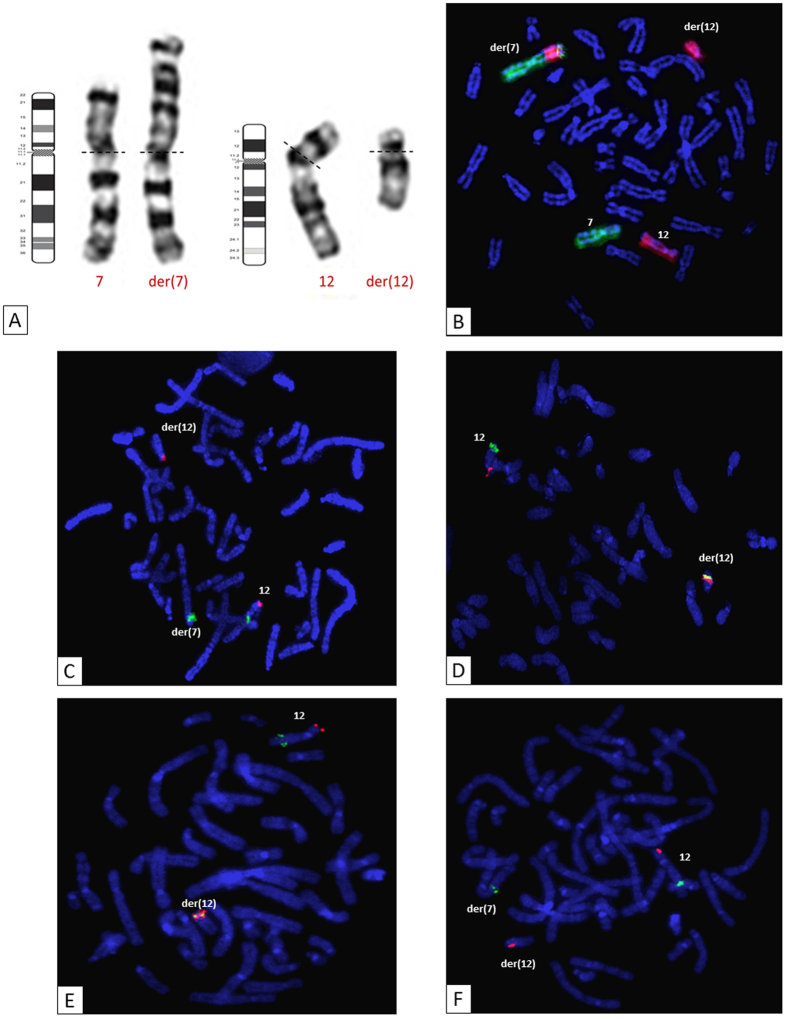
Conventional cytogenetic analysis. (**A**) Chromosome 7 and 12 G-banded karyotype and ideogram representation, showing structural abnormalities on chromosomes 7 and 12 [der(7) and der(12), respectively]. (**B**) Whole-chromosome painting probes for chromosomes 7 (green) and 12 (red) demonstrated the insertion of part of chromosome 12 into the short arm of chromosome 7. (**C**) Insertion event: FISH with BAC probes specific for the 12p13.31 (RP4-751H1; red) and 12q13.13 (RP11-1136G11; green) regions. (**D**) Inversion event: FISH with BAC probes specific for the 12p13.1 (RP11-180M15; red) and 12q24.11 (RP11-457O10; green) regions. (**E,F**) The fifth breakpoint: FISH with BAC probes specific for the 12p13.31 (RP11-444J21; red) and 12q23.2 (RP11-321F8; green) regions.

Conventional cytogenetic analyses revealed a CCR involving structural abnormalities of chromosomes 7 and 12. The patient carried an abnormal chromosome 7, with an abnormally long short arm, and an abnormal chromosome 12, both arms of which were shortened (Fig. 1A).

We used whole chromosome-specific DNA probes for chromosomes 7 and 12 to characterize the chromosomal mechanism of the CCR (Fig. 1B). An analysis of the hybridization pattern observed revealed that a region of chromosome 12 had inserted into the short arm of one chromosome 7 (Fig. 1B). This insertion event was confirmed by the use of a probe binding to the 12q13.13 region (Fig. 1C). This probe hybridized to the long arm of the normal chromosome 12 and the short arm of the der(7) (derivative chromosome 7), confirming the insertion event (Fig. 1C). The insertion was shown to be inverted (Supplementary Table 1). In addition to the rearrangement mentioned above, chromosome 12 displayed aberrant G banding and had a short arm that was abnormally short (Fig. 1A). All the subtelomeric ends were in their usual positions on chromosomes 7 and 12 and their derivatives (data not shown). FISH analysis was performed with two probes, one binding to the short arm of chromosome 12 in the 12p13.1 region and the other binding to the long arm of chromosome 12 in the 12q24.11 region: the 12q24.11 probe signal was detected in its normal position on the normal and der(12) (derivative chromosome 12), whereas the 12p13.1 probe signal was located on the long arm of chromosome 12 (Fig. 1D). These results indicate that a pericentric inversion of the der(12) had occurred in addition to the insertion event.

**Figure 2:**
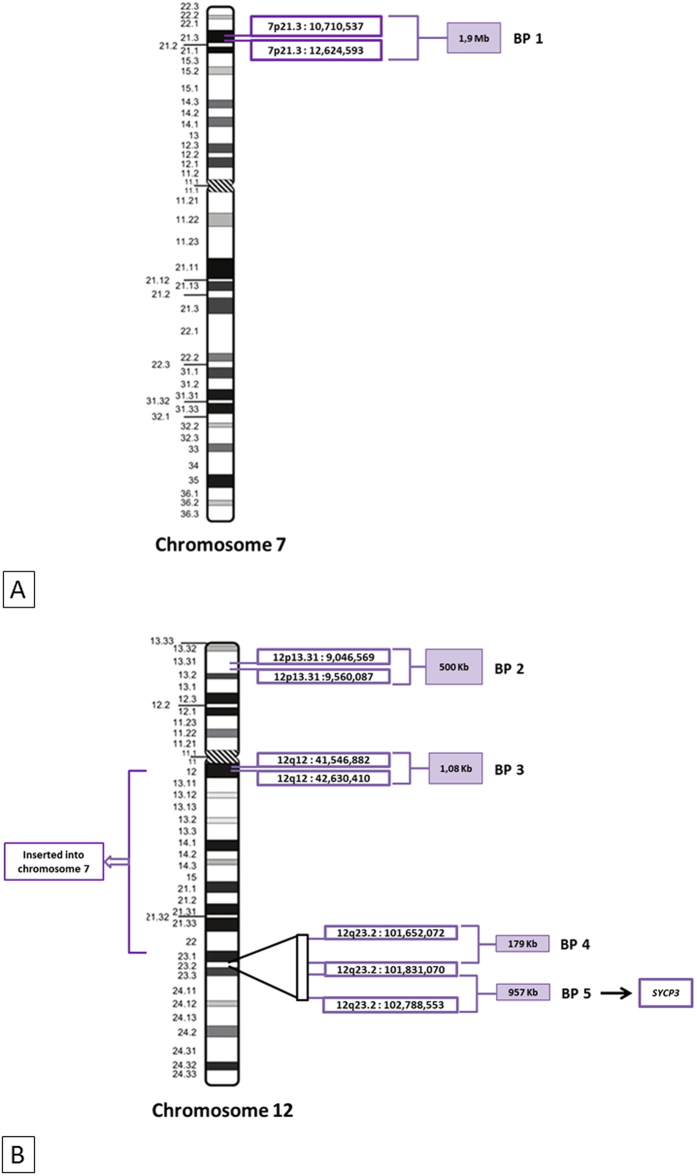
Location of the complex chromosomal rearrangement breakpoints. Ideograms for chromosome 7 (**A**) and chromosome 12 (**B**), showing the five breakpoints identified by successive FISH hybridizations (see Supplementary Table 1). BP5 contained the *SYCP3* gene. BP: breakpoint.

Two breakpoints were predicted in the 12q23.2 region, rather than a common breakpoint for the two events (Fig. 2). Using a probe binding to the chromosome 12:101, 652, 072–101, 831, 070 interval of the 12q23.2 region, we detected two distinct signals for these probes on metaphases (Fig. 1E,F and Supplementary Table 1). These results ruled out the existence of a common breakpoint, instead suggesting that there was a fifth breakpoint.

We used a molecular cytogenetic approach based on FISH to define the location of the breakpoints involved in the CCR. The results and the BAC probes used for these experiments are listed in Supplementary Table I. In total, five breakpoints were implicated in this CCR (Fig. 2A and B). We identified three breakpoints for the insertion event on der(7) and der(12): one breakpoint on the short arm of der(7), corresponding to the 7p21.3 chromosome band (chr7:10, 710, 537–12, 624, 593), and two breakpoints on the long arm of der(12), at 12q12 (chr12:41, 546, 882–42, 630, 410) and 12q23.2 (chr12:101, 652, 072–101, 831, 070) (Supplementary Table 1 and Fig. 2). The inversion gave rise to two other breakpoints on der(12), in regions 12p13.31 (chr12:9, 046, 569–9, 560, 087) and 12q23.2 (chr12:101, 830, 070–102, 788, 553) (Supplementary Table 1). The chromosomal formula (ISCN 2013) was: 46, XY, inv(12)(p13.31q23.2), ins(7;12)(p21.3;q23.2q12). Gene mapping in these regions identified a candidate gene in the 12q23.2 region: *SYCP3*, an essential structural component of the synaptonemal complex involved in the synapsis, recombination and segregation of meiotic chromosomes.

Array-CGH was performed to assess the presence of cryptic imbalance at the CCR breakpoints or elsewhere in the genome. This analysis revealed no relevant DNA copy number variation that might account for the azoospermia phenotype. Thus, the patient carried a balanced complex chromosomal rearrangement. The karyotypes of his parents were normal, so the chromosomal rearrangement observed in the patient had occurred *de novo*. Furthermore, molecular analysis revealed the absence of a microdeletion in the AZF a/b/c regions.

### Generation of induced pluripotent stem cells (iPSCs) from the patient’s erythroblasts

**Figure 3:**
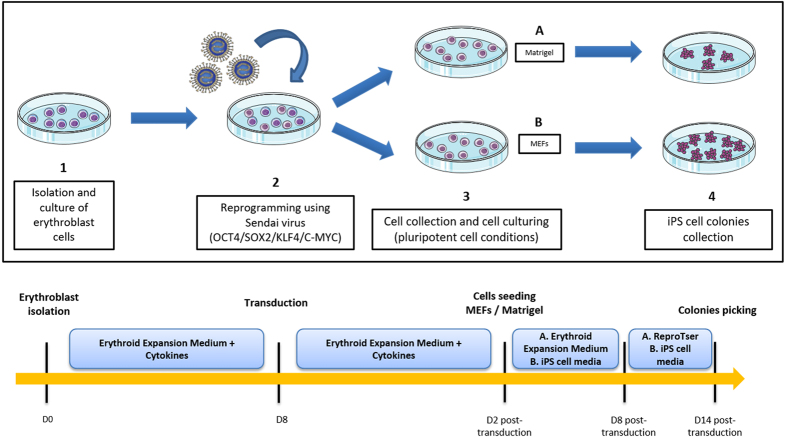
Timeline and result of *in vitro* generation of human induced pluripotent stem cells on MEFs and Matrigel. C-Myc: v-myc avian myelocytomatosis viral oncogene homolog; D: day; iPSCs: induced pluripotent stem cells; KLF4: Kruppel-like factor 4 (gut); MEF: mouse embryonic fibroblast; OCT4 (also called POU5F1): POU class 5 homeobox 1; SOX2: SRY-box 2. The figure was produced, in part, by using Servier Medical Art, (www.servier.com/Powerpoint-image-bank).

Peripheral blood mononuclear cells (PBMCs) were purified from blood collected from the patient, and cultured in specific medium to induce erythroblast differentiation. After nine days of expansion, FACS analysis with antibodies known to bind erythroid cell surface markers revealed that about 60% of the cells were CD71^+^ and GpA^+^ erythroblasts. We generated patient-specific iPSCs, by transducing erythroblasts with Sendai viruses expressing the four reprogramming factors and culturing them on mouse embryonic fibroblast feeder cells or a Matrigel-coated plate. The cells began to adopt an iPSC-like morphology 15 days after transduction. Colonies were collected and expanded. Larger numbers of iPSC colonies were generated from culture on MEFs than in Matrigel conditions. Moreover, in the long term, growth on MEF feeder cells resulted in more stable iPSC lines. Five clones were evaluated for pluripotency. The protocol is summarized in Fig. 3.

### Pluripotency marker expression

**Figure 4:**
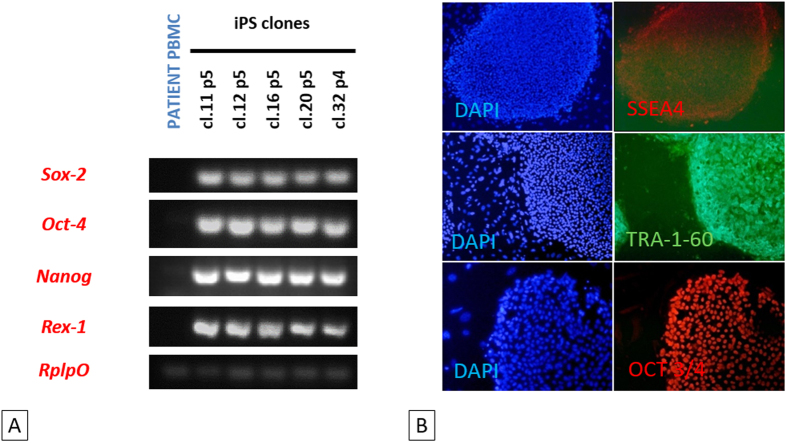
Endogenous expression of pluripotency-related markers by iPSCs. (**A**) RT-PCR analysis for detection of the pluripotency markers *SOX2, OCT4, NANOG* and *REX-1*. All the patient iPSC clones tested expressed these genes. See full-length gels in Supplementary Figure 2. (**B**) Immunofluorescence staining of three stem cell proteins (SSEA4, TRA-1-60 and OCT3/4) in patient iPS clones 12 and 32. Both clones 12 and 32, at passages 5 and 4, respectively, expressed the three pluripotency markers used.

We investigated the pluripotency of the five selected iPSC clones, by analyzing the expression of the endogenous pluripotency marker genes *SOX2, OCT4, NANOG* and *REX-1* by RT-PCR. *RPL*P0 gene expression was used as an internal control for this assay. The pluripotency markers were strongly expressed in all five iPSC clones but not in the primary cells (PBMCs) from the patient (Fig. 4A) We also performed immunofluorescence assays, in which we observed the specific staining of SSEA4, TR-1-60 and OCT3/4 pluripotency markers in iPSC colonies (Fig. 4B). Thus, the iPSC-like clones derived from the infertile patient expressed conventional pluripotency markers, indicating that they had been successfully reprogrammed.

### Evaluation of pluripotency and of the differentiation potential of patient-specific iPS clones

**Figure 5:**
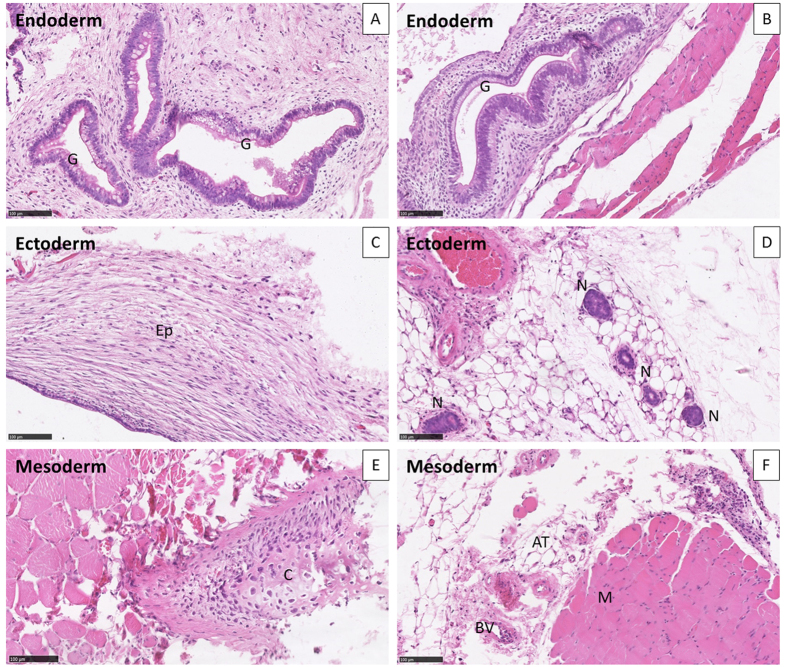
Germ cell layer components within teratomas. The differentiation, at passage 6, of iPSCs into ectoderm, endoderm and mesoderm was evaluated on whole sections stained with hematoxylin and eosin, for iPS clones 12 (**A**,**C**,**E**) and 32 (**B**,**D**,**F**). Both clones displayed structures representing the three lineages: (**A**,**B**) endoderm. (**C**,**D**) ectoderm. (**E**,**F**) mesoderm. Hematoxylin and eosin stain, ×20. AT, adipose tissue; BV, blood vessel; C, cartilage; G, gut epithelial tissue; Ep, keratin-containing epidermal tissue; M, striated muscle; N, neural tissue.

We explored the teratoma-forming potential of two patient-specific iPS clones (cl-12 and cl-32), and carried out a histological analyses of tumors derived from these clones. These teratoma assays provided a clear evaluation of the impact on differentiation and proliferation of patient iPSCs *in vivo* over a period of several weeks. For the teratoma assay, colonies were picked during early passages (p6) and injected into muscle. Patient-specific iPS clones 12 and 32 produced teratomas (Fig. 5).

Histological analysis of the tumors produced showed that these cells had differentiated into endodermal (a, b), ectodermal (c, d) and mesodermal (e, f) tissues, in the form of glandular gut-like epithelium (G), epidermal tissue (Ep), neural tissue (N), large cartilaginous areas (C), muscle (M), adipocyte tissue (AT) and blood vessels (BV) (Fig. 5). The tissues were well-differentiated, without malignancy, in all the structures observed.

### Patient-specific iPSC karyotype and array-CGH

The karyotypes of iPSC cl-12 and cl-32 were realized at passages 11 and 12 respectively, to assess the stability of the rearrangement involving chromosomes 7 and 12 in the genome of the patient. The same CCR was identified, indicating that the primary cells of the patient had not undergone additional chromosomal alterations during the reprogramming protocol (Supplementary Fig. 1).

We evaluated the genomic stability of iPSCs further, by performing 1 M array-CGH analyses in parallel on cl-12 (p11) and cl-32 (p12), and on lymphocytes from the patient. These analyses provided no evidence of additional pathogenic genomic losses or gains.

## Discussion

The patient described here had non-obstructive azoospermia and, therefore displayed an abnormal progression of the succession of spermatogenetic stages. First, conventional cytogenetics studies identified a CCR, which was characterized by fluorescence *in situ* hybridization and array-CGH. The complex rearrangement involved chromosomes 7 and 12, with five breakpoints (7p13.3, 12p13.31, 12q12, 12q23 and 12q23). The short arm of der(7) contained inserted inverted material from the long arm of der(12), and a pericentric inversion had occurred in the resulting der(12). Second, we successfully generated patient-specific iPSCs from erythroblasts in a context of chromosomal abnormalities. *In vitro* tests on the iPSCs demonstrated the expression of endogenous pluripotency markers and teratoma assays confirmed the pluripotency of the stem cells *in vivo*.

**Figure 6:**
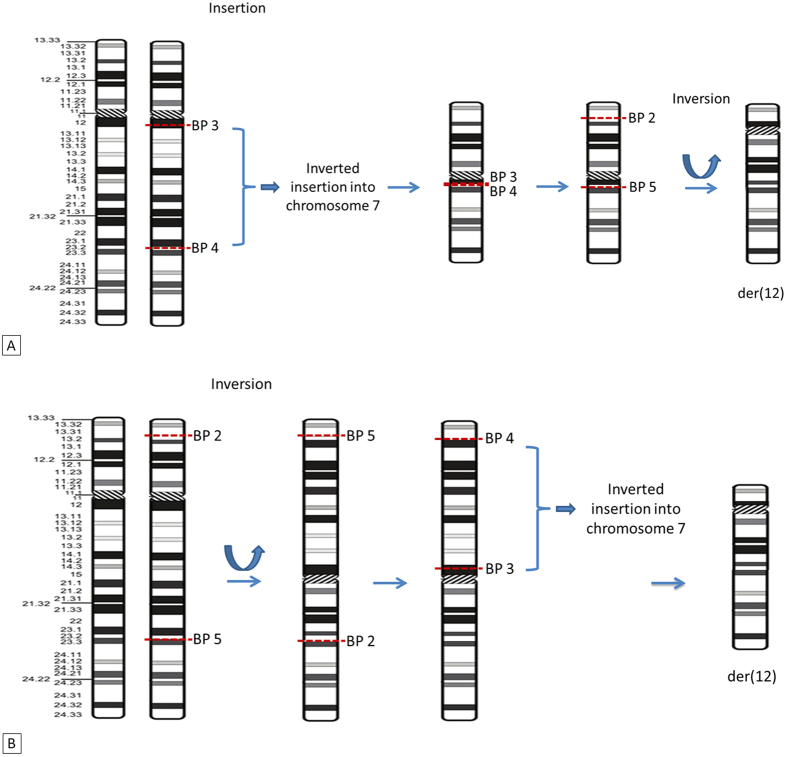
Two hypothetical chromosomal mechanisms accounting for the complex chromosomal rearrangement in the patient. (**A**) With the inversion event occurring first. (**B**) With the insertion event occurring first. BP: breakpoint.

Instead of a common breakpoint for the two events, the 12q23.2 region contained two breakpoints. Using a probe binding to the 12q23.2 region, we identified a fifth breakpoint, explaining the mechanism underlying the CCR (Fig. 1E and F). If the insertion and inversion events had a common breakpoint in the 12q23.2 region, located between 101, 652, 072–101, 831, 070 (RP11-321F8), then two signals would have been obtained, either on the long arm of der(12) or on the short arm of der(7). A representation of the rearranged chromosomes and a proposed sequence of events that may have led to this abnormal karyotype are shown in Fig. 6.

Chromosomal abnormalities leading to oligozoospermia or azoospermia are among the most common causes of male infertility. Abnormalities associated with chromosome 7 and 12 have been reported before in patients with non-obstructive azoospermia, including a paracentric inversion of chromosome 7 and a pericentric inversion of chromosome 12^34^,^35^, and translocations involving chromosome 12^36^. However, none of these previously reported cases had breakpoints [inv (12) (p12q12); inv (7)(q22-31)] in common with our patient [inv(12)(p13.31q23.2), ins(7;12)(p21.3;q23.2q12)]. CCRs, including translocations, inversions, insertions, and other structural chromosomal abnormalities, can have important consequences for the pairing of meiotic chromosomes during pachytene. In mammals, this process is essential for progression through meiosis, and is achieved through a recombination mechanism initiated by DNA double-strand breaks (DSBs)^37^. These breaks are essential for normal male gametogenesis^37^.

Normal chromosomes form bivalents during this stage. The presence of a rearrangement may lead to multivalent formation and the mechanical disruption of chromosome pairing. In this configuration, some or all of the chromosomes remain unpaired, and, therefore, unsynapsed^37^. The repair of meiotic DSBs requires homologous synapsis. Asynapsis results in the persistence of DSBs, silencing genes in the unsynapsed chromosomal segment, potentially resulting in spermatogenetic failure if the genes silenced are involved in this process^37^,^38^,^39^. Indeed, infertility may result from chromosomal defects leading to pachytene asynapsis or the disruption of genes involved in spermatogenesis.

Not all structural rearrangements of chromosomes impair sperm production or function and are associated with infertility or subfertility phenotypes^40^. A few exceptional cases of the male inheritance of CCRs have been documented^40^,^41^. Thus, in exceptional cases, the CCRs in male carriers may be able to form appropriate pachytene configurations during meiosis. The mechanism underlying asynapsis-related male-specific meiotic arrest is not fully understood. CCR rearrangements are frequently associated with reproductive failure, with a high risk of chromosomal abnormalities in offspring, recurrent spontaneous abortions and infertility^42^,^43^. Thus, chromosomal rearrangements do not necessarily lead to meiotic arrest due to synapsis problems. Disruptions of essential meiotic genes may, therefore, also result in infertility.

The locations of the breakpoints in the rearrangement are also thought to affect the fertility of the carrier. The sequences of genes involved in male gametogenesis and residing in these chromosomal breakpoint regions, and those of their regulatory elements, may be disrupted or deregulated. The generation of fusion transcripts by chromosome rearrangement may also contribute to azoospermia. Large numbers of genes have been associated with infertility^8^,^20^. Many of these genes were characterized in mouse models^8^. Meiotic genes are highly conserved in the evolution of species and their disruption has provided strong evidence of their negative impact on fertility. In our case, breakpoint mapping for the balanced CCR identified a candidate gene, *SYCP3*. This gene encodes a structural component of the axial and lateral parts of the synaptonemal complex. The synaptonemal complex mediates the pairing and synapsis of homologous chromosomes at the pachytene stage, and several reports have shown mutations of this gene to be associated with azoospermia in man and susceptibility to pregnancy loss^44^,^45^,^46^. Thus, the interruption of *SYCP3* may have altered expression of the protein, leading to synaptonemal complex dysfunction. As a result, meiosis was initiated but not completed as suggested by the testicular biopsy. Indeed, histological analysis showed that germline cells in seminiferous tubules were mostly at the spermatocyte stage, with only a few reaching the spermatid stage. As a consequence, no sperm was found in the ejaculate.

Cytogenetic and molecular analysis, Y-chromosome microdeletion screening, FISH techniques and other genetic methods, such as array-CGH and next-generation sequencing, have provided considerable insight into the genetics of infertility^12^,^47^,^48^. However, our understanding of the genetic causes of male infertility remains limited. With the emergence of iPSC technology, cells from patients can be easily reprogrammed, and then directed to differentiate into the desired lineage. These iPSCs contain the genetic heritage of the patient and, thus, constitute an excellent model for exploring specific diseases in humans^49^.

In this study, we used non-integrative Sendai viruses producing defined factors to reprogram erythoblasts from our azoospermic patient to obtain pluripotent cells. The iPSC colonies obtained had a typical embryonic stem cell (ESC)-like morphology and expressed pluripotency markers. These iPSCs formed teratomas with all three germ layers, demonstrating their pluripotency *in vivo*. These results provide a reliable demonstration of the generation of fully pluripotent iPSCs from cells carrying a complex chromosomal rearrangement.

In recent years, some laboratories have managed to impose a germline fate on ESCs and iPSCs, mostly in mice^28^. Since 2011, the differentiation of human iPSCs into haploid male germ cells has been achieved^50^,^51^,^52^,^53^. Several studies have also demonstrated extensive expression of the synaptonemal complex. Ramathal and his team recently demonstrated the capacity of human-derived AZF-deficient iPSCs to differentiate into primordial germ-like cells *in vitro*^31^. This finding highlights the potential and progress of *in vitro* gametogenesis research.

The patient-specific iPSCs derived in this report are the first *in vitro* genetic model to be described for studies of the mechanisms by which chromosomal aberrations affect spermatogenesis in humans.

The ability of these cells to differentiate into germ cells will be assessed based on published protocols^31^,^53^,^54^. For *in vitro* strategies, patient-iPSC lines stably carrying a *VASA*-GFP *reporter* construct may be useful for monitoring the generation of PG-like cells during embryonic body differentiation or monolayer differentiation approaches. The expression of specific germ cell genes such as *VASA, DAZL, BLIMP1, NANOS3, STRA8* and of genes associated with more advanced differentiation, such as *SCP1* (meiotic prophase), *ACR* (spermatid to spermatozoa), and *PRM1* (post-meiotic), will be evaluated by RT-qPCR. The results of these studies will help to determine the stage at which the blockade occurs within the patient’s cells^28^,^31^,^53^,^54^. In the case of our patient, if *SYCP3* is the only gene responsible for infertility, then the xenotransplantation of patient-specific iPSCs into mouse testis should induce PGC-like development^31^. An appropriate *in vitro* protocol, reproducing the development of human germ cells *in vivo*, has also been shown to lead to the differentiation into PGC-like, and the transduction of iPSCs with a lentiviral vector carrying the *SYCP3* gene might be able to repair the defect.

The successful derivation of infertile patient-specific iPSCs reported here highlights the real potential of this approach for the *in vitro* modeling of gametogenesis in the context of a complex chromosomal rearrangement. Further analyses should demonstrate the utility of these cells for deciphering the molecular mechanisms underlying genetically driven male infertility.

## Materials and Methods

### Patient

The patient was 38 years old and consulted for infertility after he and his partner had been trying to conceive for two years. The patient was the first child of unrelated parents, and he had four brothers and five sisters whose fertility status could not be determined because of their personal situations (they were younger and not actively trying to procreate). Clinical examination excluded obstruction of the genital tract but revealed marked bilateral atrophy of the gonads, with a testicular volume of only 7 cm^3^ (normal range: 20–25 cm^3^). Semen was collected after a requested five-day period of abstinence. Two spermograms performed eight months apart revealed azoospermia. Testicular doppler ultrasound and ultrasound scans of the deep genital tract showed hypervascularization of the prostate, with calcification of the central zone, slight differential thickening of the pelvic walls, but without obstruction, and non-retentive vesicles. Laboratory tests revealed hormonal dysregulation, with a low serum concentration of inhibin B (36 pg/mL; normal range: 80–270 pg/mL) and a high serum concentration of FSH (15.5 IU/L; normal range: 1.4–10 IU/L). The serum concentration of LH was normal (7.9 IU/L; normal range: 1.4–8 IU/L). Bilateral testicular biopsy was performed and histological analysis showed maturation arrest in all the seminiferous tubules mostly at the spermatocyte stage and more rarely at the spermatid stage. Thus, meiosis was initiated but not completed. In addition, testicular Leydig’s cell hyperplasia was observed. Genetic analyses, including karyotype, fluorescence *in situ* hybridization, Y chromosome microdeletion and array-CGH, were required to determine the cause of the infertility. Informed consent for genetic analyses was obtained from the patient, in accordance with local ethics guidelines and regulations (Assistance Publique – Hôpitaux de Paris). The patient provided written, informed consent in accordance with the guidelines and regulations of French law (Code Civil, Article 16–10). All methods were performed in accordance with French law (Décret n°2008-31 du 4 avril 2008). The experimental protocol was approved by the Assistance Publique – Hôpitaux de Paris institutional committee.

### Conventional cytogenetic analysis

Standard chromosomal analyses were performed on cultured peripheral lymphocytes from the patient and his parents and on derived iPSC clones, by standard procedures [G-banding with trypsin using Giemsa (GTG); R-banding after heat denaturation and Giemsa (RHG)].

### Fluorescence *in situ* hybridization (FISH)

FISH analyses were performed on metaphase spreads of lymphocytes from the patient. The following probes were used, in accordance with the manufacturer’s recommendations: whole-chromosome painting (WCP) probes specific for chromosomes 7 and 12 (Kreatech), centromeric probes specific for chromosomes 7 and 12 (Vysis) and subtelomeric probes specific for chromosomes 7 and 12 (Cambio). Bacterial artificial chromosome (BAC) clones specific for the chromosome 12 short arm (RP4-751H1, RP11-444J21, RP11-35C21 and RP11-346G18 located at 12p13.31; RP11-281L3 located at 12p13.2; RP11-180M15 located at 12p13.1; and RP11-459D22 located at 12p12.3), long arm (RP11-282A3, RP11-35C21, RP11-351C21 and RP11-95K16 located at 12q12; RP11-474P2 and RP3-432E18 located at 12q13.11; RP11-1136G11 located at 12q13.13; RP11-290I21 located at 12q14.2; RP11-444B24 located at 12q15; RP11-228G3, RP11-54P10, RP11-11 M4 and RP11-147C4 located at 12q21.33; RP11-536G4 located at 12q22; RP11-155C14 and RP11-434E3 located at 12q23.1; RP11-321F8, RP11-210L7 and RP11-553C19 located at 12q23.2; RP11-205I24 and RP11-43D4 located at 12q23.3; RP11-457O10 located at 12q24.11; and RP1-315L5 located at 12q24.12); and the chromosome 7 short arm (RP11-505D17, RP5-1008N9 and RP11-139O17 located at 7p21.3; RP11-79G16 and RP11-547G15 located at 7p21.2) were used (Bluegnome).

### DNA extraction

Genomic DNA was isolated from the patient’s peripheral blood and from derived iPSCs, with the Maxwell^®^ 16 Blood DNA Purification Kit (Promega, Biotech). The concentration and quality of the extracted DNA were evaluated with a NanoDrop ND-1000 spectrophotometer (NanoDrop Technologies). The extracted DNA was used for Y-chromosome AZF microdeletion and array-CGH analysis.

### Y-chromosome AZF region screening

Y-chromosome microdeletions in the AZFa, AZFb, and AZFc regions were analyzed through routine molecular diagnosis of Y chromosome abnormalities in accordance with the EAA/EMQN Guidelines. We used a sequence-tagged site (STS)–PCR approach to analyze microdeletions of the Y chromosome. We selected 28 STS, corresponding to the three different AZF loci, for the analysis. The STS tested were sY84 for AZFa, sY95, sY97, sY169, sY102, sY105, sY109 for the interval between AZFa and AZFb, sY113, sY115, sY117, sY124, sY130, sY134, sY136, sY143, sY142 for AZFb, sY152, sY232, sY156, sY240, sY148, sY249, sY204, sY208, sY254, sY269, sY158 for AZFc, and sY160 for the heterochromatic distal Yq region.

### Oligonucleotide-based array-comparative genomic hybridization (array-CGH)

The genomic imbalances in lymphocytes from the patient and in derived iPS clones 12 and 32 at passages p11 and p12, respectively, were investigated by array-CGH with 1M oligonucleotide arrays (Agilent Technologies). Array hybridization was performed according to the manufacturer’s instructions. In brief, 0.5 μg of genomic DNA was fluorescently labeled with the Agilent Genomic DNA labeling kit PLUS (Agilent Technologies). Male human genomic DNA was used as a reference. Cy5-dUTP patient DNA and sex-matched reference DNA labeled with Cy3-dUTP were denatured and preannealed with Cot-1 DNA and Agilent blocking reagent before hybridization for 40 h, with rotation at 20 rpm, at 65 °C in a rotating hybridization oven (Agilent Technologies). The slides were then washed and scanned on an Agilent Microarray Scanner. The captured images were processed with Feature Extraction 10.7.3.1 software and data analysis was performed with Cytogenomics 3.0.1.1 software. Copy number variations (CNVs) were considered significant if they were defined by three or more contiguous oligonucleotides spanning at least 2 kb and were not identified in the Database of Genomic Variants. The Genome Browser used to analyze gene content was hg19, Build37 (http://genome.ucsc.edu/).

### PCR analysis of gene expression

Total RNA was isolated from iPSCs with the NucleoSpin RNA II kit (Macherey Nagel), in accordance with the manufacturer’s protocol. We reverse-transcribed 1 μg of each RNA sample to generate cDNA, with an iScript cDNA Synthesis Kit (Bio-Rad). RT-PCR was performed with the GoTaq DNA Polymerase kit (Promega). PCR products were separated by electrophoresis in a 1% agarose gel, and analyzed with the Gel Doc 2000 System (Bio-Rad). RT-PCR for the 5′ coding region was performed with primers specific for *OCT4* (sense primer 5′-AGCGAACCAGTATCGAGAAC-3′ and reverse primer 5′-TTACAGAACCACACTCGGAC-3′), *SOX2* (sense primer 5′-AGCTACAGCATGATGCAGGA-3′ and reverse primer 5′-GGTCATGGAGTTGTACTGCA-3′), *NANOG* (sense primer 5′-TGAACCTCAGCTACAAACAG -3′ and reverse primer 5′-TGGTGGTAGGAAGAGTAAAG-3′), *TBP* (sense primer 5′-CTCACAGGTCAAAGGTTTAC-3′ and reverse primer 5′-GCTGAGGTTGCAGGAATTGA-3′), *REX1* (sense primer 5′-CAGTCCAGCAGGTGTTTGC-3′ and reverse primer 5′-GCATTCTATGTAACAGTCTGAGA-3′).

### Peripheral blood mononuclear cell (PBMC) purification and erythroblast expansion

PBMCs were isolated from whole blood by Ficoll density gradient separation, with SepMate kit tubes, according to the manufacturer’s instructions (SepMate™, StemCell Technologies). We used 150,000 PBMCs in total for erythroblast expansion in a serum-free erythroid expansion medium from Stemcell Technologies. After eight to nine days, the size of the erythoblast population was estimated by flow cytometry with anti-CD71 and erythroid-specific anti-glyphorin-A antibodies, both from e-Biosciences (eBioscience).

### Induced pluripotent stem cell (iPSC) generation by Sendai virus-mediated gene transfer

Once the erythoblast population had been expanded from the patient’s PBMCs, we used a Sendai virus (Life Technologies) for cell reprogramming.

In brief, we incubated 150,000 erythroblasts in StemSpan containing erythroid cytokines for 24 h. Sendai viruses encoding OCT3/4, SOX2, KLF4, and C-MYC pluripotency factors were added, in accordance with the manufacturer’s instructions, in the presence of StemSpan medium containing erythroid expansion factors. Two days later, the cells were used to seed cultures on MEF feeder cells in the presence of one volume of StemSpan medium with erythroid cytokines and 2 volumes of iPS medium supplemented with bFGF, or to seed one volume of StemSpan medium with erythroid cytokines and 2 volumes of ReproTeSR in Matrigel-coated plates. The medium was progressively replaced, ending with 100% iPSC medium supplemented with bFGF, or 100% ReproTeSR medium for cells on Matrigel. The generation of iPSC colonies was monitored daily, by checking for morphological changes. Colonies began to appear two weeks after transduction and were picked during the 3^rd^ and 4^th^ weeks.

### Immunostaining and immunofluorescence microscopy

For immunofluorescence assays, cells were fixed by incubation in 4% paraformaldehyde for 30 minutes, permeabilized by incubation in 0.2% Triton X-100 for 30 minutes and blocked by incubation with 3% BSA and 5% donkey serum in PBS (Chemicon). The cells were then incubated overnight with antibodies directed against OCT3/4 (1/100) (Abcam ab19857), SSEA-4-AF555 (1:50) (BD 560218) and Tra-1-60-AF488 (1:100) (Miltenyi Biotec). The cells were washed and Alexa Fluor 555-conjugated (1:500) donkey anti-rabbit IgG (Life Technologies) was added for the detection of Oct3/4.

### Teratoma formation

Confluent undifferentiated iPSCs were treated with 1 mg/ml collagenase IV (Roche), resuspended in a mixture (2:1:1) of DMEM (PAA), Matrigel (Becton Dickinson) and collagen (Life Technologies) and injected intramuscularly into seven- to 10-week-old immunodeficient mice. Teratomas formed within eight to 12 weeks. They were excised and fixed. Histological analysis was performed on sections stained with hematoxylin-eosin. All animals were used according to protocols approved by the local animal ethics advisory committee, registered with the French research ministry and in accordance with French national regulation (national transposition of European directive 2010/63/CE). All animal experiments were approved by the Commissariat à l'énergie atomique et aux énergies alternatives (CEA), 92265 Fontenay-aux-Roses, France.

## Additional Information

**How to cite this article**: Mouka, A. *et al*. Induced pluripotent stem cell generation from a man carrying a complex chromosomal rearrangement as a genetic model for infertility studies. *Sci. Rep.* **7**, 39760; doi: 10.1038/srep39760 (2017).

**Publisher's note:** Springer Nature remains neutral with regard to jurisdictional claims in published maps and institutional affiliations.

## Supplementary Material

Supplementary Figures and Table

## References

[b1] Hull M. G. . Population study of causes, treatment, and outcome of infertility. British medical journal (Clinical research ed.) 291, 1693–1697 (1985). 3935248 10.1136/bmj.291.6510.1693 PMC1418755

[b2] Sun F. . Abnormal progression through meiosis in men with nonobstructive azoospermia. Fertility and Sterility 87 (2007). 10.1016/j.fertnstert.2006.07.1531 17140569

[b3] Cocuzza M., Alvarenga C. & Pagani R. The epidemiology and etiology of azoospermia. Clinics (Sao Paulo, Brazil) 68 Suppl 1, 15–26 (2013). 10.6061/clinics/2013(Sup01)03 PMC3583160 23503951

[b4] Nakamura Y. . Chromosomal variants among 1790 infertile men. International journal of urology: official journal of the Japanese Urological Association 8, 49–52 (2001). 11240825 10.1046/j.1442-2042.2001.00242.x

[b5] Dong Y. . Copy number variations in spermatogenic failure patients with chromosomal abnormalities and unexplained azoospermia. Genetics and molecular research: GMR 14, 16041–16049, doi: 10.4238/2015.December.7.17 (2015). 26662397

[b6] Marquet V. . Double deletion of a chromosome 21 inserted in a chromosome 22 in an azoospermic patient. Clinical case reports 3, 757–761, doi: 10.1002/ccr3.313 (2015). 26401282 PMC4574793

[b7] Egozcue S. . Human male infertility: chromosome anomalies, meiotic disorders, abnormal spermatozoa and recurrent abortion. Hum Reprod Update 6, 93–105 (2000). 10711834 10.1093/humupd/6.1.93

[b8] Matzuk M. M. & Lamb D. J. The biology of infertility: research advances and clinical challenges. Nature medicine 14, 1197–1213, doi: 10.1038/nm.f.1895 (2008). PMC3786590 18989307

[b9] Manvelyan M. . Forty-eight new cases with infertility due to balanced chromosomal rearrangements: detailed molecular cytogenetic analysis of the 90 involved breakpoints. Int J Mol Med 19, 855–864 (2007). 17487417 10.3892/ijmm.19.6.855

[b10] Wang L. . Abnormal meiotic recombination with complex chromosomal rearrangement in an azoospermic man. Reprod Biomed Online 30, 651–658, doi: 10.1016/j.rbmo.2015.02.015 (2015). 25892501

[b11] Liu X. G., Hu H. Y., Guo Y. H. & Sun Y. P. Correlation between Y chromosome microdeletion and male infertility. Genetics and molecular research: GMR 15, doi: 10.4238/gmr.15028426 (2016). 27323142

[b12] Guediche N. . Array comparative genomic hybridization analysis of small supernumerary marker chromosomes in human infertility. Reprod Biomed Online 24, 72–82, doi: 10.1016/j.rbmo.2011.08.014 (2012). 22116069

[b13] Armanet N., Tosca L., Brisset S., Liehr T. & Tachdjian G. Small Supernumerary Marker Chromosomes in Human Infertility. Cytogenetic and genome research 146, 100–108, doi: 10.1159/000438718 (2015). 26398339

[b14] Brisset S. . Cytogenetic, molecular and testicular tissue studies in an infertile 45, X male carrying an unbalanced (Y;22) translocation: case report. Hum Reprod 20, 2168–2172, doi: 10.1093/humrep/dei034 (2005). 15845593

[b15] Martin R. H. Cytogenetic determinants of male fertility. Hum Reprod Update 14, 379–390, doi: 10.1093/humupd/dmn017 (2008). 18535003 PMC2423221

[b16] Song S. H., Chiba K., Ramasamy R. & Lamb D. J. Recent advances in the genetics of testicular failure. Asian J Androl 18, 350–355, doi: 10.4103/1008-682x.178857 (2016). 27048782 PMC4854078

[b17] Mahadevaiah S. K., Setterfield L. A. & Mittwoch U. Pachytene pairing and sperm counts in mice with single Robertsonian translocations and monobrachial compounds. Cytogenetics and cell genetics 53, 26–31 (1990). 2323225 10.1159/000132889

[b18] Miklos G. L. Sex-chromosome pairing and male fertility. Cytogenetics and cell genetics 13, 558–577 (1974). 4218807 10.1159/000130307

[b19] Cooke H. J. & Saunders P. T. Mouse models of male infertility. Nature reviews. Genetics 3, 790–801, doi: 10.1038/nrg911 (2002). 12360237

[b20] Massart A., Lissens W., Tournaye H. & Stouffs K. Genetic causes of spermatogenic failure. Asian J Androl 14, 40–48, doi: 10.1038/aja.2011.67 (2012). 22138898 PMC3735159

[b21] Wernig M. . *In vitro* reprogramming of fibroblasts into a pluripotent ES-cell-like state. Nature 448, 318–324 (2007). 17554336 10.1038/nature05944

[b22] Okita K., Ichisaka T. & Yamanaka S. Generation of germline-competent induced pluripotent stem cells. Nature 448, 313–317 (2007). 17554338 10.1038/nature05934

[b23] Maherali N. . Directly reprogrammed fibroblasts show global epigenetic remodeling and widespread tissue contribution. Cell stem cell 1, 55–70 (2007). 18371336 10.1016/j.stem.2007.05.014

[b24] Dimos J. T. . Induced pluripotent stem cells generated from patients with ALS can be differentiated into motor neurons. Science New York, N.Y 321, 1218–1221 (2008). 10.1126/science.1158799 18669821

[b25] Park I. H. . Disease-specific induced pluripotent stem cells. Cell 134, 877–886 (2008). 18691744 10.1016/j.cell.2008.07.041 PMC2633781

[b26] Siller R., Greenhough S., Park I. H. & Sullivan G. J. Modelling human disease with pluripotent stem cells. Current gene therapy 13, 99–110 (2013). 23444871 10.2174/1566523211313020004 PMC3785403

[b27] Takahashi K. & Yamanaka S. Induction of Pluripotent Stem Cells from Mouse Embryonic and Adult Fibroblast Cultures by Defined Factors. Cell 126, 663–676 (2006). 16904174 10.1016/j.cell.2006.07.024

[b28] Mouka A., Tachdjian G., Dupont J., Drevillon L. & Tosca L. *In Vitro* Gamete Differentiation from Pluripotent Stem Cells as a Promising Therapy for Infertility. Stem Cells Dev 25, 509–521, doi: 10.1089/scd.2015.0230 (2016). 26873432

[b29] Abed S. . Transplantation of Macaca cynomolgus iPS-derived hematopoietic cells in NSG immunodeficient mice. Haematologica 100, e428–431, doi: 10.3324/haematol.2015.127373 (2015). 26088930 PMC4591782

[b30] Sterneckert J. L., Reinhardt P. & Scholer H. R. Investigating human disease using stem cell models. Nature reviews. Genetics 15, 625–639, doi: 10.1038/nrg3764 (2014). 25069490

[b31] Ramathal C. . Fate of iPSCs Derived from Azoospermic and Fertile Men following Xenotransplantation to Murine Seminiferous Tubules. Cell Reports 7, 1284–1297 (2014). 24794432 10.1016/j.celrep.2014.03.067 PMC4283769

[b32] Ma Y. . Aberrant gene expression profiles in pluripotent stem cells induced from fibroblasts of a Klinefelter syndrome patient. J Biol Chem 287, 38970–38979, doi: 10.1074/jbc.M112.380204 (2012). 23019320 PMC3493938

[b33] Shimizu T. . Derivation of integration-free iPSCs from a Klinefelter syndrome patient. Reproductive medicine and biology 15, 35–43, doi: 10.1007/s12522-015-0213-9 (2016). 26709348 PMC4686545

[b34] Ghorbel M. . Pericentric inversion of chromosom 12 [Inv (12) (p12q12)] associated with idiopathic azoospermia in one infertile Tunisian man. Biochem Biophys Res Commun 432, 472–474, doi: 10.1016/j.bbrc.2013.01.110 (2013). 23399567

[b35] Ichioka K., Yoshimura K., Honda T., Takahashi A. & Terai A. Paracentric inversion of chromosome 7(q22-31) associated with nonobstructive azoospermia. Fertil Steril 83, 455–456, doi: 10.1016/j.fertnstert.2004.06.070 (2005). 15705391

[b36] Li L. . Mapping breakpoints of a familial chromosome insertion (18, 7) (q22.1; q36.2q21.11) to DPP6 and CACNA2D1 genes in an azoospermic male. Gene 547, 43–49, doi: 10.1016/j.gene.2014.06.007 (2014). 24937803

[b37] Homolka D., Jansa P. & Forejt J. Genetically enhanced asynapsis of autosomal chromatin promotes transcriptional dysregulation and meiotic failure. Chromosoma 121, 91–104, doi: 10.1007/s00412-011-0346-5 (2012). 22002499 PMC3260437

[b38] Schoenmakers S. . Increased frequency of asynapsis and associated meiotic silencing of heterologous chromatin in the presence of irradiation-induced extra DNA double strand breaks. Dev Biol 317, 270–281, doi: 10.1016/j.ydbio.2008.02.027 (2008). 18384767

[b39] Burgoyne P. S. & Baker T. G. Meiotic pairing and gametogenic failure. Symposia of the Society for Experimental Biology 38, 349–362 (1984). 6545729

[b40] Cai T. . A de novo complex chromosomal rearrangement with a translocation 7;9 and 8q insertion in a male carrier with no infertility. Human Reproduction 16, 59–62 (2001). 11139537 10.1093/humrep/16.1.59

[b41] Grasshoff U. . A complex chromosomal rearrangement with a translocation 4;10;14 in a fertile male carrier: ascertainment through an offspring with partial trisomy 14q13–>q24.1 and partial monosomy 4q27–>q28 [corrected]. Cytogenetic and genome research 103, 17–23, doi: 76282 (2003). 15004458 10.1159/000076282

[b42] Pellestor F. . Meiotic segregation of complex reciprocal translocations: direct analysis of the spermatozoa of a t(5;13;14) carrier. Fertil Steril 95, 2433 e2417–2422, doi: 10.1016/j.fertnstert.2011.01.159 (2011). 21367411

[b43] Madan K., Nieuwint A. W. & van Bever Y. Recombination in a balanced complex translocation of a mother leading to a balanced reciprocal translocation in the child. Review of 60 cases of balanced complex translocations. Human genetics 99, 806–815 (1997). 9187678 10.1007/s004390050453

[b44] Gurkan H., Aydin F., Kadioglu A. & Palanduz S. Investigation of mutations in the synaptonemal complex protein 3 (SYCP3) gene among azoospermic infertile male patients in the Turkish population. Andrologia 45, 92–100, doi: 10.1111/j.1439-0272.2012.01317.x (2013). 22670862

[b45] Miyamoto T. . Azoospermia in patients heterozygous for a mutation in SYCP3. Lancet (London, England) 362, 1714–1719, doi: 10.1016/s0140-6736(03)14845-3 (2003). 14643120

[b46] Stouffs K., Lissens W., Tournaye H., Van Steirteghem A. & Liebaers I. SYCP3 mutations are uncommon in patients with azoospermia. Fertil Steril 84, 1019–1020, doi: 10.1016/j.fertnstert.2005.04.033 (2005). 16213863

[b47] Hofherr S. E., Wiktor A. E., Kipp B. R., Dawson D. B. & Van Dyke D. L. Clinical diagnostic testing for the cytogenetic and molecular causes of male infertility: the Mayo Clinic experience. J Assist Reprod Genet 28, 1091–1098, doi: 10.1007/s10815-011-9633-6 (2011). 21912980 PMC3224174

[b48] Dada R., Gupta N. P. & Kucheria K. Cytogenetic and molecular analysis of male infertility: Y chromosome deletion during nonobstructive azoospermia and severe oligozoospermia. Cell biochemistry and biophysics 44, 171–177, doi: 10.1385/cbb:44:1:171 (2006). 16456245

[b49] Trounson A., Shepard K. A. & DeWitt N. D. Human disease modeling with induced pluripotent stem cells. Curr Opin Genet Dev 22, 509–516, doi: 10.1016/j.gde.2012.07.004 (2012). 22868174

[b50] Panula S. . Human germ cell differentiation from fetal- and adult-derived induced pluripotent stem cells. Human Molecular Genetics 20, 752–762 (2011). 21131292 10.1093/hmg/ddq520 PMC3024045

[b51] Medrano J. V., Ramathala C., Nguyena H. N., Simon C. & Reijo Pera R. A. Divergent RNA-Binding Proteins, DAZL and VASA, Induce Meiotic Progression in Human Germ Cells Derived *In Vitro*. Stem Cells 30, 441–451 (2012). 22162380 10.1002/stem.1012 PMC3695740

[b52] Easley C. A. t. . Direct differentiation of human pluripotent stem cells into haploid spermatogenic cells. Cell Rep 2, 440–446, doi: 10.1016/j.celrep.2012.07.015 (2012). 22921399 PMC3698576

[b53] Eguizabal C. . Complete Meiosis from Human Induced Pluripotent Stem Cells. Stem Cells 29, 1186–1195 (2011). 21681858 10.1002/stem.672

[b54] Zhou G. B., Meng Q. G. & Li N. *In vitro* derivation of germ cells from embryonic stem cells in mammals. Molecular reproduction and development 77, 586–594, doi: 10.1002/mrd.21187 (2010). 20575083

