# Supplementary material for: Induced pluripotent stem cell generation from a man carrying a complex chromosomal rearrangement as a genetic model for infertility studies
Source: Sci Rep. 2017 Jan 3;7:39760. doi: 10.1038/srep39760 (PMC5206619; doi:10.1038/srep39760)
Supplement: Supplementary Figures and Table [file srep39760-s1.pdf]

Induced pluripotent stem cell generation from a man carrying a complex chromosomal rearrangement as a genetic model for infertility studies

Mouka Aurélie [1, 2], Izard Vincent [3], Tachdjian Gérard[1, 2], Brisset Sophie[1, 2], Yates Frank[4], Mayeur Anne[1], Drévillon Loïc[1], Jarray Rafika[4], Leboulch Philippe[5], Maouche-Chrétien Leila[5*], Tosca Lucie[1,2*]

SUPPLEMENTARY FIGURE AND TABLE LEGENDS

Supplementary Figure 1: iPSC karyotype.

G-banded karyotypes for patient iPSC clones 12 (A) and 32 (B), at passages 11 and 12, respectively. The karyotypes showed no additional chromosomal abnormalities after reprogramming or early during culture *in vitro*.

Supplementary Figure 2: Full-length gels of RT-PCR analysis for detection of the pluripotency markers *SOX2*, *OCT4*, *NANOG* and *REX-1*.

Supplementary Table 1: BAC DNA probes used to characterize the CCR and to localize breakpoints.

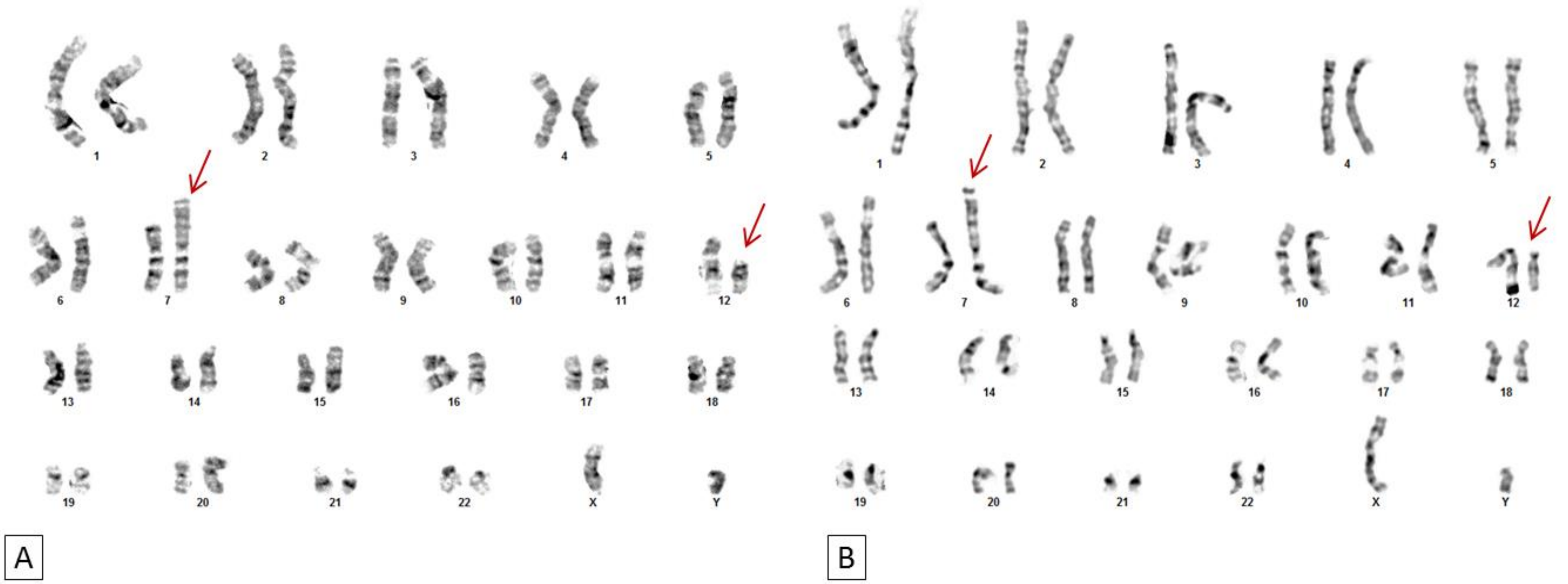

Supplementary Figure 1

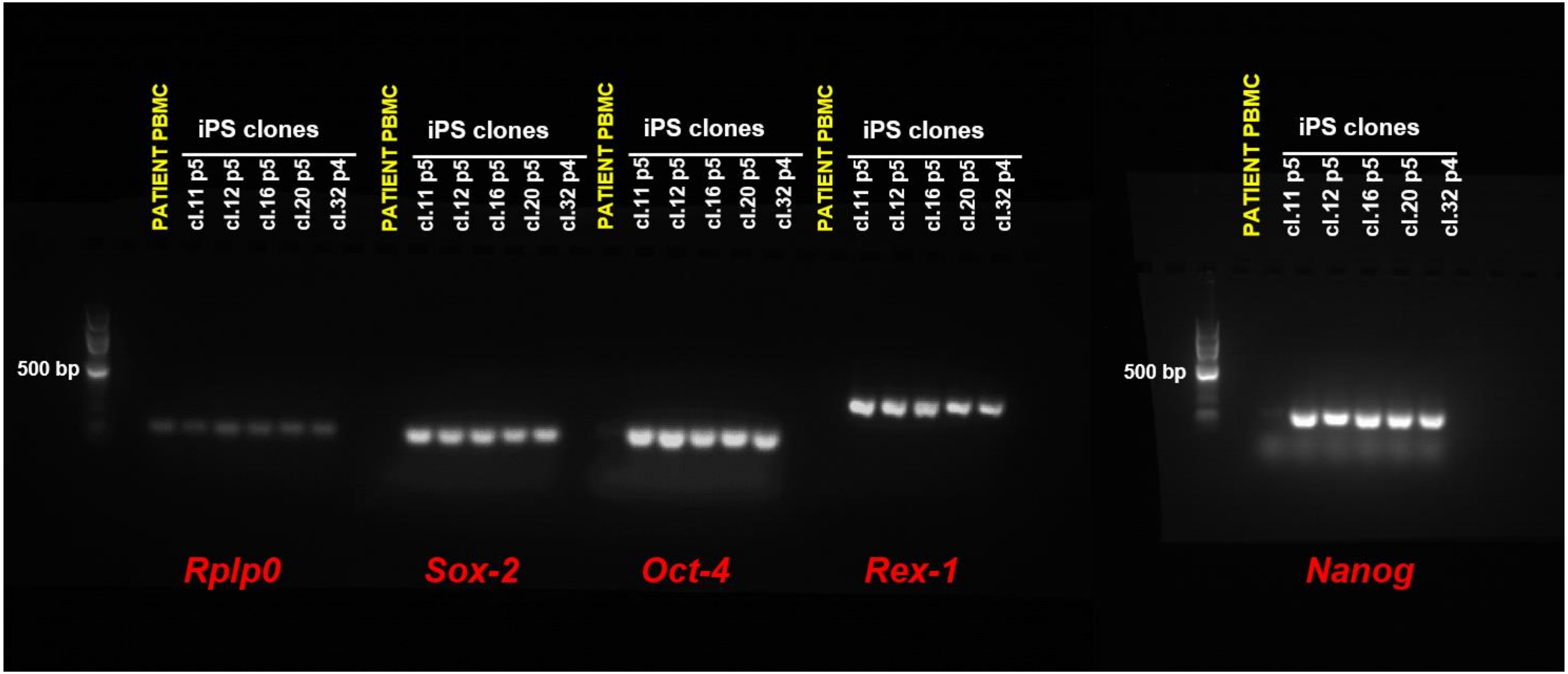

Supplementary Figure 2

Bluegnome Probe ID	Cytoband	Start (hg19)	Stop (hg19)	Observed localization
**Insertion event breakpoint on der(7) short arm: BP 1**				
RP11-505D17	7p21.3	7,981,234	8,159,394	In place on der(7) distal to the inserted fragment
RP5-1008N9	7p21.3	10,538,372	10,710,537	
RP11-139O17	7p21.3	12,624,593	12,789,972	In place on der(7) proximal to the inserted fragment
RP11-79G16	7p21.2	13,819,281	13,969,290	
RP11-547G15	7p21.2	14,896,877	15,089,802	
**Inversion event breakpoint on der(12) short arm: BP 2**				
RP4-751H1	12p13.31	6,023,233	6,158,569	In place on der(12)
RP11-444J21	12p13.31	7,716,948	7,889,198	
RP11-35C21	12p13.31	8,893,328	9,046,569	
RP11-346G18	12p13.31	9,560,087	9,739,128	Located on der(12) long arm
RP11-281L3	12p13.2	10,077,711	10,252,598	
RP11-180M15	12p13.1	12,723,966	12,880,857	
RP11-459D22	12p12.3	18,464,044	18,666,277	
**Insertion event breakpoint on der(12) long arm: BP 3**				
RP11-282A3	12q12	40,163,328	40,355,100	Located on der(12) long arm
RP11-35C21	12q12	41,375,116	41,546,882	
RP11-351C21	12q12	42,630,410	42,786,155	Located on der(7) short arm
RP11-95K16	12q12	44,243,419	44,412,355	
RP11-474P2	12q13.11	46,591,227	46,813,901	
RP3-432E18	12q13.11	47,871,644	48,012,712	
RP11-1136G11	12q13.13	53,460,231	53,616,994	
RP11-290I21	12q14.2	64,567,977	64,719,641	
RP11-444B24	12q15	68,454,646	68,645,525	
RP11-228G3	12q21.31	84,782,677	84,869,464	
RP11-54P10	12q21.33	89,008,495	89,169,334	
RP11-11M4	12q21.33	89,568,301	89,744,451	
RP11-147C4	12q21.33	92,488,040	92,638,222	
RP11-536G4	12q22	96,082,538	96,288,903	
RP11-155C14	12q23.1	99,999,685	100,153,237	
**Insertion event breakpoint on der(12) long arm: BP 4 and BP 5**				
RP11-434E3	12q23.1	100,567,515	100,763,514	Located on de(7) short arm
RP11-321F8	12q23.2	101,652,072	101,831,070	Located on der(7) short arm and on der(12) short arm
RP11-210L7	12q23.2	102,788,553	102,958,357	In place on der(12)
RP11-553C19	12q23.2	103,585,618	103,758,359	
RP11-205I24	12q23.3	103,966,166	104,123,564	
RP11-43D4	12q23.3	104,775,247	104,954,152	
RP11-457O10	12q24.11	108,982,119	109,165,140	
RP1-315L5	12q24.12	111,856,680	112,010,700	

Supplementary Table 1
